# Microbial Community Succession and Nutrient Cycling Responses following Perturbations of Experimental Saltwater Aquaria

**DOI:** 10.1128/mSphere.00043-19

**Published:** 2019-02-20

**Authors:** Holly M. Bik, Alexandra Alexiev, Sabreen K. Aulakh, Lakshmi Bharadwaj, Jennifer Flanagan, John M. Haggerty, Sarah M. Hird, Guillaume Jospin, Jenna M. Lang, Laura A. Sauder, Josh D. Neufeld, Andrew Shaver, Akshay Sethi, Jonathan A. Eisen, David A. Coil

**Affiliations:** a UC Davis Genome Center, University of California—Davis, Davis, California, USA; b Department of Biology, San Diego State University, San Diego, California, USA; c Department of Molecular and Cell Biology, University of Connecticut, Storrs, Connecticut, USA; d Department of Biology, University of Waterloo, Waterloo, Ontario, Canada; e Department of Evolution and Ecology, University of California—Davis, Davis, California, USA; f Department of Medical Microbiology and Immunology, University of California—Davis, Davis, California, USA; University of Wisconsin—Madison

**Keywords:** 16S rRNA gene, bacteria, community succession, metabarcoding, saltwater aquarium, water chemistry

## Abstract

Although aquaria are common features of homes and other buildings, little is known about how environmental perturbations (i.e., tank cleaning, water changes, addition of habitat features) impact the diversity and succession of aquarium microbial communities. In this study, we sought to evaluate the hypotheses that newly established aquaria show clear microbial successional patterns over time and that common marine aquarium-conditioning practices, such as the addition of ocean-derived “live rocks” (defined as any “dead coral skeleton covered with crustose coralline algae” transferred into an aquarium from open ocean habitats) impact the diversity of microbial populations as well as nitrogen cycling in aquaria. We collected water chemistry data alongside water and sediment samples from two independent and newly established saltwater aquaria over a 3-month period. Microbial communities in samples were assessed by DNA extraction, amplification of the 16S rRNA gene, and Illumina MiSeq sequencing. Our results showed clear and replicable patterns of community succession in both aquaria, with the existence of multiple stable states for aquarium microbial assemblages. Notably, our results show that changes in aquarium microbial communities do not always correlate with water chemistry measurements and that operational taxonomic unit (OTU)-level patterns relevant to nitrogen cycling were not reported as statistically significant. Overall, our results demonstrate that aquarium perturbations have a substantial impact on microbial community profiles of aquarium water and sediment and that the addition of live rocks improves nutrient cycling by shifting aquarium communities toward a more typical saltwater assemblage of microbial taxa.

**IMPORTANCE** Saltwater aquaria are living systems that support a complex biological community of fish, invertebrates, and microbes. The health and maintenance of saltwater tanks are pressing concerns for home hobbyists, zoos, and professionals in the aquarium trade; however, we do not yet understand the underlying microbial species interactions and community dynamics which contribute to tank setup and conditioning. This report provides a detailed view of ecological succession and changes in microbial community assemblages in two saltwater aquaria which were sampled over a 3-month period, from initial tank setup and conditioning with “live rocks” through subsequent tank cleanings and water replacement. Our results showed that microbial succession appeared to be consistent and replicable across both aquaria. However, changes in microbial communities did not always correlate with water chemistry measurements, and aquarium microbial communities appear to have shifted among multiple stable states without any obvious buildup of undesirable nitrogen compounds in the tank environment.

## Abstract (precis)

Saltwater aquaria are living systems that support a complex biological community of fish, invertebrates, and microbes. The health and maintenance of saltwater tanks are pressing concerns for home hobbyists, zoos, and professionals in the aquarium trade; however, we do not yet understand the underlying microbial species interactions and community dynamics which contribute to tank setup and conditioning. This report provides a detailed view of ecological succession and changes in microbial community assemblages in two saltwater aquaria which were sampled over a 3-month period, from initial tank setup and conditioning with “live rocks” through subsequent tank cleanings and water replacement. Our results showed that microbial succession appeared to be consistent and replicable across both aquaria. However, changes in microbial communities did not always correlate with water chemistry measurements, and aquarium microbial communities appear to have shifted among multiple stable states without any obvious buildup of undesirable nitrogen compounds in the tank environment.

## INTRODUCTION

Studies of microbial ecology of the built environment focus on the distribution and composition of microbial communities sampled from air and surfaces of engineered structures (1). Such work has described microbial communities in hospitals (2–4), universities (5), residential homes and offices (6–8), and drinking water and plumbing systems (9, 10) and has been aimed at gaining a broad overview of the factors that play a role in structuring such microbial communities. Consequently, this information may one day help define and identify “healthy” versus “sick” engineered environments. Freshwater and saltwater aquaria represent subsets of the engineered environment, serving as mesocosms with conditions intended to mimic the ecology of a natural system. Aquarium health and maintenance are critical considerations for zoos and public aquaria, fish breeders, pet stores, and hobbyists. The aquarium industry has a global reach, with trade chains for animals and products that span six continents (11). Most aquaria are established and maintained according to industry protocols and recommendations, with adjustments made on the basis of visual inspections (e.g., checking for signs of disease and for algal growth) and chemical testing (e.g., pH, nitrite, and ammonia). Aquarists face the challenge of establishing and maintaining dynamic communities, not just of fish, coral, and other invertebrates, but also of the microbial communities necessary for maintaining water quality and the health of target fauna. To date, few studies have attempted to track the changes in microbial community composition that are relevant for assessing aquarium health (12). Very little is known about the reproducibility of microbial succession within a newly established aquarium and its resistance to disturbance, which together may have implications for the corresponding natural freshwater or marine systems.

An important consideration for aquarium setup and maintenance is the removal of ammonia and nitrite, which are produced via animal excretion and decaying organic matter and can be toxic to aquatic life (13, 14). Ammonia accumulations exceeding 0.1 mg liter^−1^ can result in stress, disease, or death for aquarium fauna, especially fish (14, 15). In a healthy aquarium, ammonia is degraded by the aerobic process of nitrification; ammonia (NH_3_) is converted to nitrite (NO_2_) via the process of ammonia oxidation, and nitrite is subsequently converted into nitrate (NO_3_) via nitrite oxidation. This process is governed by several distinct groups of microorganisms, collectively referred to here as “nitrogen-transforming” taxa. Initial ammonia oxidation is mediated by ammonia-oxidizing bacteria (AOB) within the *Betaproteobacteria* (e.g., *Nitrosomonas* spp.) or *Gammaproteobacteria* (e.g., *Nitrosococcus* spp. [16]), as well as ammonia-oxidizing archaea (AOA) within the phylum *Thaumarchaeota* (e.g., Nitrosopumilus maritimus [17]). Subsequently, nitrite is oxidized by nitrite-oxidizing bacteria (NOB), which belong to diverse phyla, such as the *Nitrospirae* (e.g., *Nitrospira* spp.), *Deltaproteobacteria* (e.g., *Nitrospina* spp.), *Alphaproteobacteria* (e.g., *Nitrobacter* spp.), and *Gammaproteobacteria* (e.g., *Nitrococcus* spp. [18]). In addition, recent work has discovered *Nitrospira* species that are capable of complete ammonia oxidation (19, 20), where a single bacterium possesses all the enzymes required for both ammonia and nitrite oxidation. Although never reported to occur within aquarium environments, ammonia may also be converted to nitrogen gas (N_2_) in the process of anaerobic ammonium oxidation (anammox), governed by bacteria within the *Planctomycetes* (21), which use nitrite (NO_2_-) or nitrate (NO_3_-) as an electron acceptor.

In order to promote the establishment of stable nitrifying biofilms in aquaria, new tanks can be inoculated with commercial bacterial inoculants (22, 23) or can be modified by addition of sediment and filter material from established aquaria. The addition of bacterial supplements can help reduce ammonia and nitrite levels compared to those seen with untreated aquaria (22), effectively reducing the “conditioning period” required for nitrification to be established in new tanks (23). Another conditioning technique involves the addition of “live rock,” defined as any “dead coral skeleton covered with crustose coralline algae” transferred into an aquarium from open ocean habitats (13). Although the size, shape, and composition of live rock can vary widely, these untreated coral skeletons appear to host microbial species capable of removing nitrogenous compounds that represent well-known chemical stressors for fish and invertebrate species (13). The microbial community involved in nitrification varies according to aquarium and water chemistry. For example, the ratios and compositions of genes belonging to AOA and AOB in freshwater differ from those in saltwater aquaria (24, 25), and shifts in dominant AOB species are correlated with measured ammonium levels (26). Thus, subtle but important shifts in microbial communities may occur, despite the outward appearance of a healthy and stable nitrogen cycle (i.e., where nitrogenous compounds are rapidly broken down and do not build up to toxic levels (13, 14).

Stability (i.e., consistent microbial processes that break down toxic ammonia and nitrite) is important for aquarium health, but it is unknown how predictable microbial succession is or how resilient it is to disturbance. Previously, replacement of 90% of the water in an aquarium system was shown to increase the diversity and evenness of microbial populations, but the results demonstrated only a weak correlation with physical and chemical measurements (12). However, that study reported only broad patterns related to a single disturbance event. In temperate lakes, microbial communities have been shown to recover and restabilize quickly following large disturbance events such as artificial mixing (27), with community recovery strongly linked to biogeochemical factors such as oxygen levels. In coastal marine systems, disturbances such as storms can prompt differential and localized responses among microbial assemblages (28), where habitat diversity and connectivity promote resistance and resilience in response to disturbance effects. Such large-scale ecological studies in natural systems are relevant for understanding observed shifts and patterns in aquarium mesocosms.

**FIG 1:**
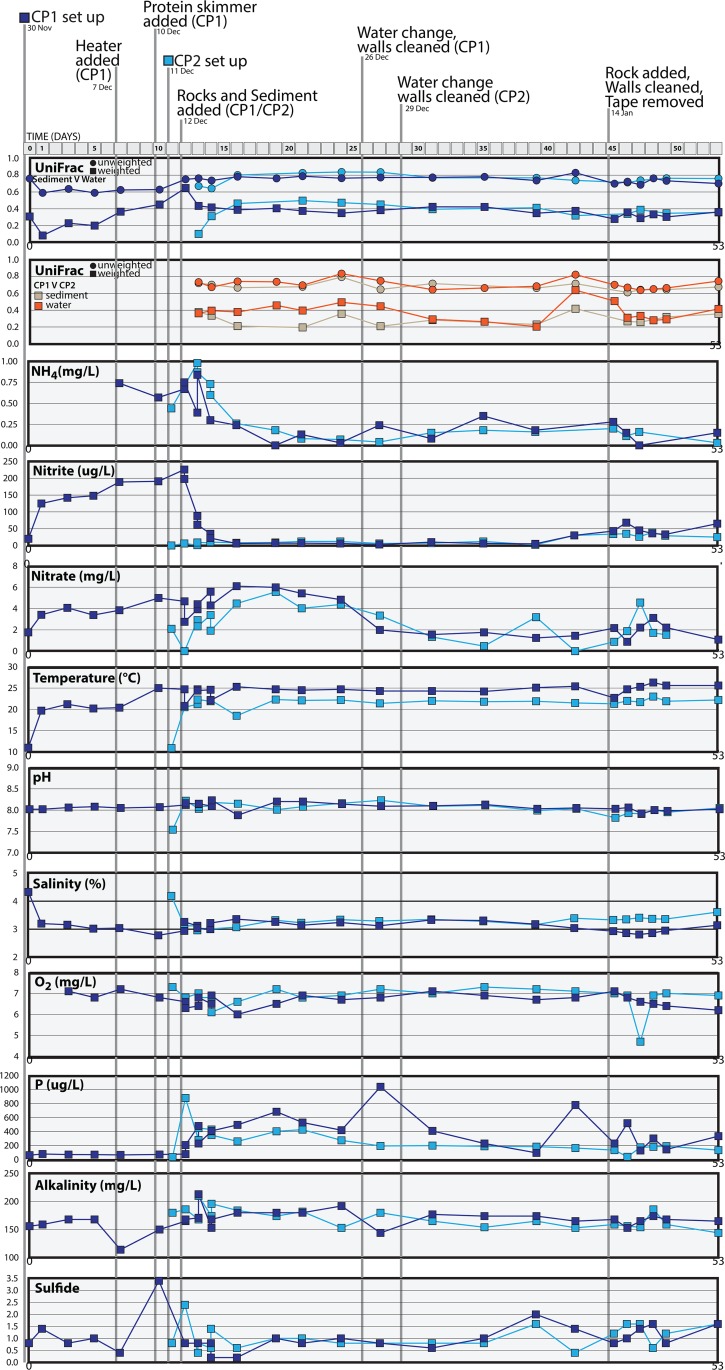
Water chemistry data, UniFrac results, and perturbations to coral pond 1 (CP1, dark blue) and coral pond 2 (CP2, light blue) experimental aquaria, displayed across time. The top panel displays UniFrac distances between sediment and water communities for each aquarium. The second panel shows UniFrac distances of microbial community 16S rRNA gene profiles between aquaria for a given substrate (sediment or water samples taken on the same date, regardless of when the aquarium was set up). The bottom 10 panels show water chemistry data over time for the two saltwater aquaria. Salinity is displayed as percent salt (refractometer) values. E coli, Escherichia coli.

Here we describe a longitudinal study focused on two independent saltwater aquaria, here referred to as coral pond 1 (CP1) and coral pond 2 (CP2). This study aimed to assess both spatial and temporal changes in microbial community composition and to characterize community shifts in response to aquarium perturbations. Water and sediment samples were collected over a 3-month period, with additional samples obtained from potential “source” habitats that came into contact with the aquaria during the experimental setup. We hypothesized that changes in microbial community composition would correlate with water chemistry measurements (Fig. 1), particularly those related to nitrogen cycling, such as changes in nitrate, nitrite, and ammonium levels. Furthermore, we hypothesized that community succession would follow similar patterns in the two independent aquaria and that the microbial communities would show distinct spatial/temporal assemblages and successional patterns within each aquarium. To test these hypotheses, we assessed the bacterial and archaeal community composition using high-throughput metabarcoding of 16S rRNA genes on the Illumina MiSeq platform. Our results demonstrate that aquarium perturbations have a substantial impact on microbial community profiles in both water and sediment locations and that the addition of habitat features such as live rocks improves nutrient cycling capacity by shifting aquarium communities toward a more typical saltwater assemblage of microbial taxa.

## RESULTS AND DISCUSSION

### Overview of aquarium microbial communities.

For simplicity, we refer to bioinformatically assigned taxonomic names of operational taxonomic units (OTUs) throughout Results and Discussion. However, we realize that (i) 16S rRNA gene OTUs are not equivalent to microbial species and (ii) taxonomic assignments of OTUs are imperfect and influenced by factors such as the completeness (or sparseness) of public sequences databases and the computational algorithms used. Thus, as much as possible, we have focused on higher-level taxonomic groupings and patterns, which are generally considered to produce more robust results in analyzing 16S rRNA gene-based OTUs.

**FIG 2:**
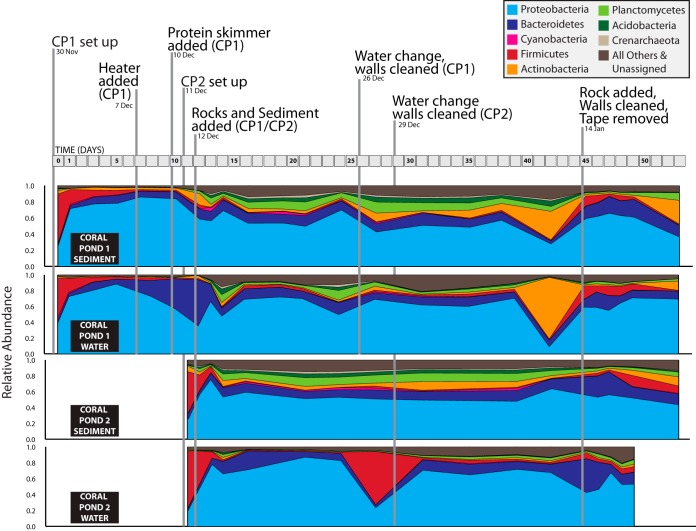
Area chart of alpha taxonomy for water and sediment samples from coral pond 1 (CP1) and coral pond 2 (CP2) experimental aquaria, plotted across time. Results are from 97% open-reference OTU picking in QIIME (singletons discarded). Taxonomic assignments for 16S rRNA gene OTUs are summarized and plotted at the phylum level, with relative abundances displayed for the eight most abundant microbial phyla.

Aquarium samples were dominated by OTUs assigned to the *Proteobacteria*, *Bacteroidetes*, *Cyanobacteria*, *Firmicutes*, *Actinobacteria*, *Planctomycetes*, *Acidobacteria*, and *Crenarchaeota* (Fig. 2). Of the OTUs classified as *Crenarchaeota*, 552 were actually *Thaumarchaeota* (as this group is lumped within the *Crenarchaeota* in the QIIME-formatted Greengenes taxonomy), with the remaining OTUs representing 62 that included classes corresponding to marine benthic group A (MBGA) and MBGB, the miscellaneous Crenarchaeota group (MCG), and the marine hydrothermal vent group (MHVG). Within this group, the Thaumarchaeota also had the highest overall relative abundance across all samples collected from CP1 and CP2 (Fig. 2). Proteobacterial OTUs comprised 20% to 80% of microbial assemblages at any time point, with relative abundances commonly >50% in both water and sediment locations (Fig. 2). *Firmicutes* OTUs represented 60% to 70% of the microbial community in water and sediment of both tanks upon initial setup (Fig. 2), but these quickly decreased in abundance over subsequent days. *Firmicutes* OTUs showed some additional spikes and fluctuations in both aquaria over time, with a substantial increase in CP2 water samples during days 25 to 30 which occurred in the absence of any aquarium perturbations (Fig. 2). *Bacteroides* OTUs were continually present in all aquarium locations, exhibiting ∼10% relative abundance at most time points in CP1 and CP2. Both the *Firmicutes* and *Bacteroides* phyla dominate the human microbiome (especially in gut habitats [29]), and the initial observed dominance of *Firmicutes* OTUs may have been due to human factors, such as the handling of water and sand upon initial tank setup. However, the consistent presence of these groups in both aquaria (as well as reports of these groups from freshwater and marine habitats [30–33]) suggests that at least some proportion of *Firmicutes* and *Bacteroides* OTUs do not correspond to human-associated taxa and likely include other species or strains that are adapted to life in aquatic systems. Other OTUs assigned to *Planctomycetes*, *Acidobacteria*, and *Actinobacteria* showed a pattern of consistent low-level relative abundances across most time points and locations, with slightly higher relative abundances in CP1 and CP2 sediment samples.

**FIG 3:**
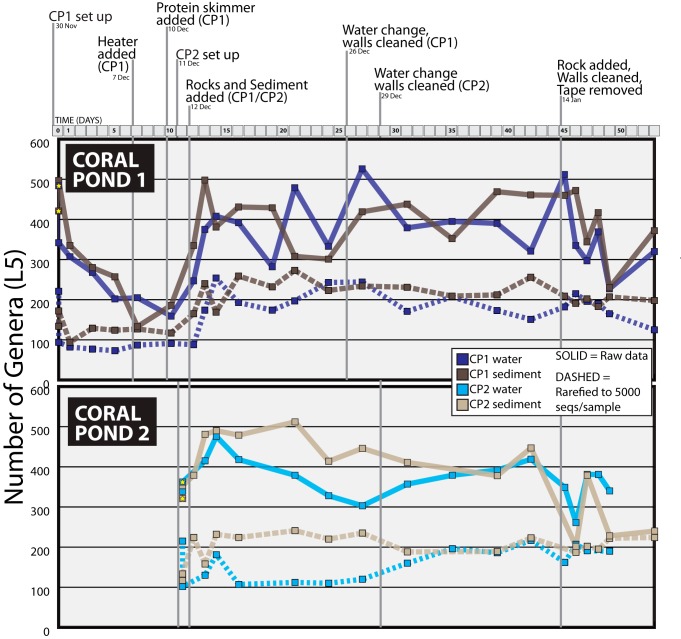
Changes in aquarium microbial community richness over time, including responses to introduced perturbations. Results from 97% open-reference OTU picking in QIIME are summarized according to genus-level taxonomy (L5, level 5 from the Greengenes taxonomic ranks). Graphs illustrate the number of bacterial/archaeal genera assigned to OTU sequences recovered across time for two independent saltwater aquaria (coral pond 1 [CP1] and coral pond 2 [CP2] water and sediment samples). Vertical lines indicate perturbations introduced during the sampling time series. On each line graph, solid colors represent overall community richness calculated from all OTUs with a minimum cluster size >2 (excluding singletons). Dashed lines represent community richness inferred for samples rarefied to 5,000 sequences per sample (with the samples with levels below that threshold discarded). Yellow stars on the far left display the genus-level OTU richness for laboratory seawater and commercial sand used for initial aquarium setup (sampled on day 0).

The first perturbation event on day 12 (addition of live rocks and sediment from an established tropical aquarium) had the most obvious overall impact on the alpha diversity levels measured for both CP1 and CP2, leading to higher genus-level diversity (Fig. 3) and an increase in the overall number of OTUs (see Fig. S1 in the supplemental material), most notably in CP1. Following this perturbation, OTUs associated with *Planctomycetes*, *Acidobacteria*, and *Crenarchaeota* showed immediate increases in relative abundance (Fig. 2); these shifts in microbial taxa were consistent across water and sediment samples from CP1 and CP2, although the relative abundance of some groups was much lower in CP2 water samples. These three phyla contain many common marine representatives, including taxa known to be involved in nitrification (21, 34, 35), thus indicating that the introduction of live rocks and transferred sediment prompted aquarium successional shifts toward microbial taxa known to be representative of saltwater habitats. The increased abundance of these major taxonomic groups was maintained across all postperturbation time points, with some additional fluctuations in abundance following the final perturbation seen on day 45.

10.1128/mSphere.00043-19.1 FIG S1 Graph of sequence reads and OTU counts obtained from coral pond 1 (CP1; panel A) and coral pond 2 (CP2, panel B) experimental aquaria over time. The first *x*-axis data point represents reads/OTUs from intake water and commercial sand used to set up aquaria; all subsequent *x*-axis data points represent days of sampling in experimental aquaria. The primary (left) *y*-axis data correspond to OTU counts (solid lines); the secondary (right) *y*-axis data correspond to sequence reads (dashed lines). Due to murkiness, no water sample was collected from CP2 on day 1; this sampling gap is depicted as a line break in panel B. Download FIG S1, PDF file, 0.8 MB. Copyright © 2019 Bik et al. 2019 Bik et al. This content is distributed under the terms of the Creative Commons Attribution 4.0 International license.

### Linking water chemistry data with observed microbial patterns.

Temporal shifts in water chemistry values indicated that the most notable changes were related to nitrogen cycling (Fig. 1). In CP1, ammonia (NH_3_-N), nitrite (NO_2_-N), and nitrate (NO_3_^-^) levels increased from the time of aquarium setup until the first perturbation on day 12, when the aquarium was “seeded” with live rocks and transferred sediment. Following this perturbation, there was an immediate reduction in ammonia and nitrite levels, presumably due to microbial activity associated with the established tank sediment and live rock biofilms. Nitrate levels also decreased following this first perturbation, although at a slightly lower rate (Fig. 1). The low initial concentrations of sulfide and phosphorus increased after the first perturbation event, although measurements of alkalinity, sulfide, and phosphorus fluctuated over the course of the experimental time series; some fluctuations appeared to be correlated with aquarium perturbations, while others occurred in isolation. In contrast, temperature, salinity, and oxygen levels were largely stable and did not fluctuate substantially over the course of the experiment, with the exception of a few outlier readings at the start of the study (e.g., during aquarium setup) and brief changes in these measurements during perturbations and tank maintenance (i.e., when new water was being added; Fig. 1). Patterns and shifts in water chemistry measurements were largely consistent across CP1 and CP2, despite the shorter time period for aquarium setup and conditioning of the CP2 experimental tank.

**FIG 4:**
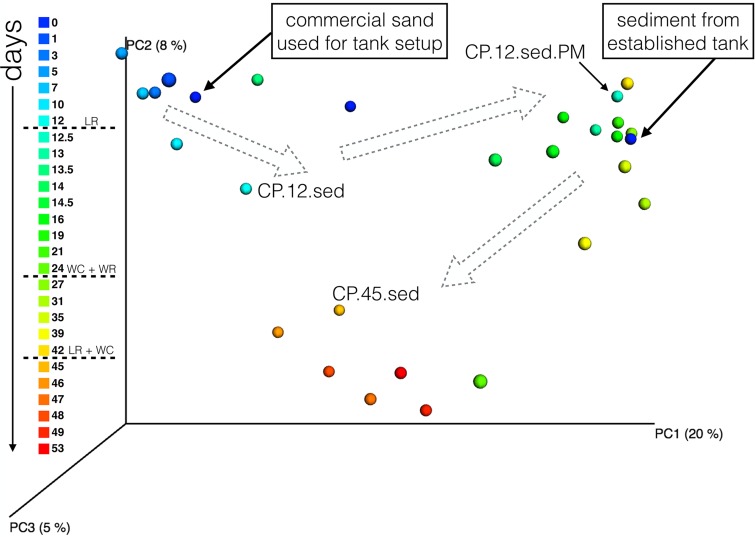
Aquarium perturbations spur large shifts in microbial beta-diversity patterns over time. Data represent results of principal-coordinate-analysis (PCoA) ordination based on unweighted UniFrac distances of microbial community 16S rRNA gene profiles, displaying sediment samples from the coral pond 1 (CP1) aquarium, with the gradient color scale illustrating sampling across time. Dotted lines on the left color legend indicate the three major perturbation events (LR = live rock addition, WC = walls cleaned, WR = water replacement). Following tank setup, microbial community assemblages showed closest similarity to those seen with the commercial sand sample used to set up the aquarium on day 0. Rocks and sediment were transferred from an established aquarium on day 12, at which point the microbial assemblage showed rapid change over the course of a few hours (CP.12.sed, collected in the morning before aquarium perturbation; CP12.sed.PM, collected in the afternoon immediately following perturbation). A second major shift in community changes occurred after the addition of live rocks (CP.45.sed onward; orange and red dots). Dotted gray arrows indicate general trends in community shifts over time. Data represent results from 97% open-reference OTU picking in QIIME (singletons discarded), rarefied at 1,000 sequences per sample.

**FIG 5:**
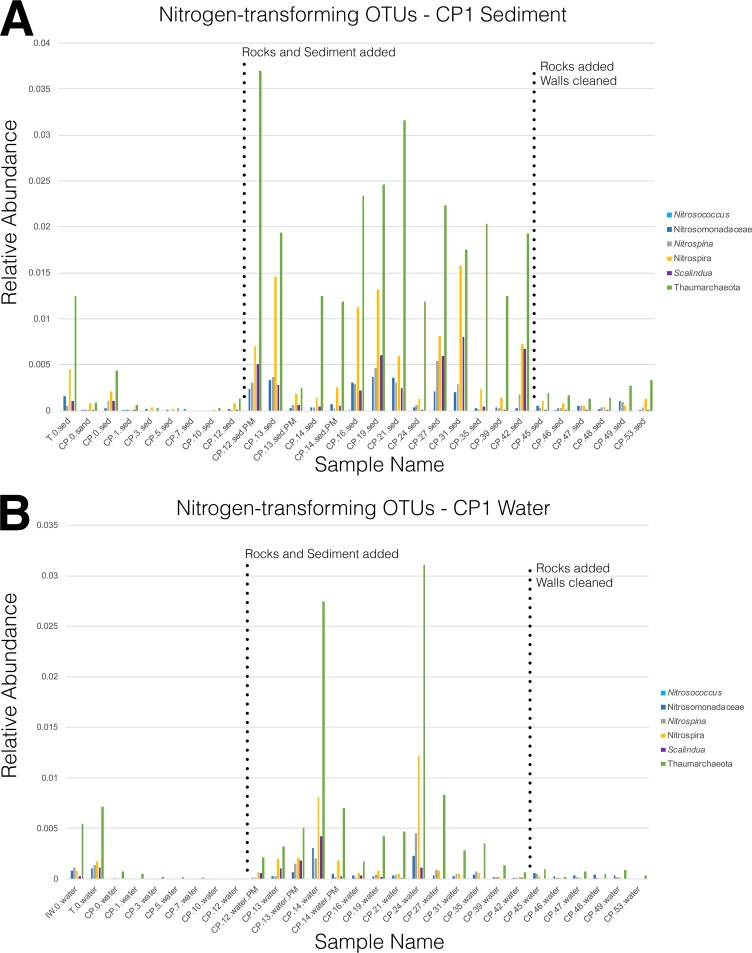
Relative abundances of putative nitrogen-transforming OTUs in the coral pond 1 (CP1) experimental aquarium. Data represent relative abundances of OTUs belonging to six different microbial groups summarized and plotted over time for sediment (A) and water (B) microhabitats. The first two bars of each panel represent baseline microbial communities (displayed for comparison) from a separate established tropical saltwater aquarium (T.0.water and T.0.sed), commercial sand used to set up aquaria (CP.0.sand), and a laboratory seawater system (IW.0.water). Microbial taxa displayed include ammonia-oxidizing bacteria (AOB; *Nitrosococcus*, *Nitrosomonadaceae*), nitrite-oxidizing bacteria (NOB; *Nitrospina*, *Nitrospira*), anaerobic ammonia-oxidizing bacteria (anammox; *Scalindua*), and ammonia-oxidizing archaea (AOA; Thaumarchaeota).

In both CP1 and CP2, the first addition of live rocks and transferred sediment (day 12) had a notable impact on microbial community structure and richness (Fig. 2 and 4). Following this first perturbation event, an increase in the diversity of microbial populations (Fig. 3) occurred at the same time as a drop in the levels of putatively human-associated bacterial groups (Fig. 2) and a simultaneous increase in the levels of characteristic nitrogen-transforming microbial taxa (Fig. 5; see also Fig. S2**)**. The most obvious and immediate change in water chemistry was a drop in ammonia levels, which may have been due to microbial ammonia oxidation governed by anammox bacteria within the *Planctomycetia* (genus *Scalindua*), AOB within the *Gammaproteobacteria* (genus *Nitrosococcus*) and *Betaproteobacteria* (family *Nitrosomonadaceae*), or AOA from the phylum Thaumarchaeota (21, 24, 25). Subsequent decreases in nitrite were likely governed by nitrite-oxidizing bacteria within the class *Nitrospira* (phylum *Nitrospirae*) and members of the *Deltaproteobacteria* genus *Nitrospina* (19, 20). These groups of known nitrogen-transforming taxa were all present in our experimental aquaria and were observed to increase in relative abundance after the first addition of live rocks and sediment on day 12 (Fig. 5; see also Fig. S2), albeit comprising a small fraction of the overall microbial community. Several groups of nitrogen-transforming microorganisms have been reported at low or variable abundances in aquarium systems (25), and the overall low relative abundances of most nitrogen-transforming taxa observed in this study were not unexpected (relative abundances of <0.04% per sample) (Fig. 5; see also Fig. S2). In particular, AOA have high substrate affinities for ammonia (24, 25), and high microbial biomass of groups such as *Thaumarchaeota* is not required due to the efficiency of the ammonia oxidation process and specific adaptations which enable these microbes to thrive even under oligotrophic conditions. The ratio of AOA to AOB taxa is known to be inversely correlated with ammonia levels, and additional physiochemical parameters and microbial community dynamics are likely to exert further influences on the relative abundances of nitrogen-transforming taxa (24, 25). Furthermore, previous studies have indicated specific habitat preferences in certain microbial groups (e.g., preferential growth of AOA on fine sponge material in biofilters [25]), and it is likely that our sampling strategy did not fully capture the spatial variability and microhabitat preferences of all nitrogen-transforming taxa. Taken together, these empirical observations build on previous scientific evidence (23) and underline the key role that live rocks and preadapted microbial communities appear to play in the conditioning and maintenance of saltwater aquaria, supporting anecdotal observations and standard practices in the aquarium industry.

10.1128/mSphere.00043-19.2 FIG S2 Relative abundances of predicted nitrogen-transforming OTUs in the coral pond 2 (CP2) experimental aquarium. Relative abundances of OTUs belonging to six different microbial groups are summarized and plotted over time for sediment (A) and water (B) microhabitats. The first two bars of each panel represent baseline microbial communities (displayed for comparison) from a separate established tropical saltwater aquarium (T.0.water and T.0.sed), commercial sand used to set up aquaria (CP2.0.sand), and a laboratory seawater system (CP2.0.intake). Microbial OTUs displayed include those identified as ammonia-oxidizing bacteria (AOB; *Nitrosococcus*, *Nitrosomonadaceae*), nitrite-oxidizing bacteria (NOB; *Nitrospina*, *Nitrospira*), anaerobic ammonia-oxidizing bacteria (anammox; *Scalindua*), and ammonia-oxidizing archaea (AOA; Thaumarchaeota). Download FIG S2, PDF file, 0.03 MB. Copyright © 2019 Bik et al. 2019 Bik et al. This content is distributed under the terms of the Creative Commons Attribution 4.0 International license.

### Impact of aquarium perturbations on microbial richness and diversity.

Although the bacterial alpha diversity of the initial inputs (commercial sand, laboratory seawater) was relatively high (300 to 500 genus-level OTUs; Fig. 3), we observed a marked decrease in microbial richness during the first 12 days of the CP1 aquarium (Fig. S1). This initial reduction of microbial OTUs was most likely due to the loss of human-associated and nonaquatic microbes (e.g., loss associated with dry commercial sand), which could not survive and reproduce in a saltwater aquarium ecosystem. However, the high levels of ammonia, nitrite, and nitrate also indicated that microbially driven nitrification was not yet optimized in CP1 prior to day 12 (Fig. 1). This hypothesis is further supported by the community similarity index data (panels with blue lines; Fig. S3), which indicate that the CP1 water and sediment communities briefly became more similar in the first ∼5 days after initial tank setup. The initial addition of live rocks and established tank sediment on day 12 resulted in an immediate increase in the number of genus-level microbial OTUs recovered from water and sediment locations in both CP1 and CP2 (Fig. 3). In CP1, the number of reported microbial genera increased from 175 to 200 (preperturbation on day 10) to 400 to 500 genera (postperturbation on day 13 onward) (Fig. 3). A consistent pattern was observed in CP2 where total microbial genera increased from ∼350 genera (preperturbation) to ∼500 genera (postperturbation) in both water and sediment locations. In terms of water chemistry data, the increase in microbial OTUs following the day 12 perturbations also coincided with a sharp drop in the levels of ammonia (NH_3_-N, free ammonia nitrogen) and nitrite (NO_2_-N) (Fig. 1), as well as with immediate fluctuations and a more gradual decrease in nitrate (NO_3_^-^) levels over the following days.

10.1128/mSphere.00043-19.3 FIG S3 Bray-Curtis, Jaccard, and Canberra beta diversity of microbial community 16S rRNA gene profiles from two experimental aquaria. The three upper panels display pairwise Bray-Curtis, Jaccard, and Canberra distances between the same substrates (sediment or water) from different aquaria (Coral pond 1 [CP1] and coral pond 2 [CP2]) at a given time point, whereas the lower three panels display distances between different substrates (sediment or water) from the same aquarium (CP1 and CP2). Download FIG S3, EPS file, 0.6 MB. Copyright © 2019 Bik et al. 2019 Bik et al. This content is distributed under the terms of the Creative Commons Attribution 4.0 International license.

The final major perturbation event on day 45 again appeared to disrupt the aquarium microbial communities. However, in contrast to the first perturbation, this resulted in a decrease in the levels of microbial OTUs across both CP1 and CP2 after day 45 (Fig. 3) as well as in a decrease in the relative abundances of several major taxonomic groups that had spiked in abundance after the initial tank perturbation on day 12. There is some indication that the microbial genera were beginning to increase in level again in both aquaria at the time that the study concluded on day 53 (Fig. 3), suggesting that further longitudinal data may have been needed to reveal the full effects of this final perturbation event. Finally, less-intensive aquarium perturbations in the middle of the study (i.e., routine water changes and wall cleaning) did not appear to impact the overall number of microbial OTUs as much as the first and last perturbations, where live rocks were added on days 12 and 45. These microbial patterns were generally consistent in the analyses of our data set using either the full OTU table (solid lines in Fig. 3) or the rarefied OTU table (a subset of 5,000 sequences per sample; dashed lines in Fig. 3).

Important goals of modern microbial ecology studies include understanding the drivers of microbial richness and diversity and identifying specific events inducing critical changes in community structure and function. Our saltwater tanks were subjected to various perturbation events as a consequence of establishing the two aquaria. All decisions related to the timing of these perturbations were made by university personnel overseeing the aquaria, based on practical constraints (e.g., time to install equipment) and previous experience with saltwater tank setup and maintenance. All microbial sampling was secondary, providing an ideal case study that mirrored typical tank setup protocols used by aquarium hobbyists and industry professionals. Perturbations included the addition of a heater and the use of a protein skimmer, water changes, tank wall cleanings, transfer of sediment from an existing tropical aquarium, and the addition of live rocks (see Fig. 2 and 3). Our results indicated that the initial sediment transfer and addition of live rock on day 12 prompted the largest shifts in microbial abundance and community structure (Fig. 3; see also Fig. S1), effectively serving as a probiotic treatment that seeded tanks with new microbial groups, altered the overall relative abundances of major taxa (Fig. 2), and increased the relative abundances of known nitrogen-transforming microbes (Fig. 5; see also Fig. S2). The postperturbation increase in the diversity of microbial populations seen on day 12 is consistent with other studies of aquarium systems, such as a study by Van Bonn et al. (12) which reported significant increases in bacterial community diversity and evenness following a 90% water change in a large saltwater tank. Similarly, the drop in the diversity of microbial populations and fluctuations of microbial assemblages following the final perturbation event on day 45 were also consistent with previous results from disturbance events introduced into aquaria and natural lake ecosystems (12, 27).

### Beta-diversity patterns reveal fine-scale temporal and spatial dynamics.

**FIG 6:**
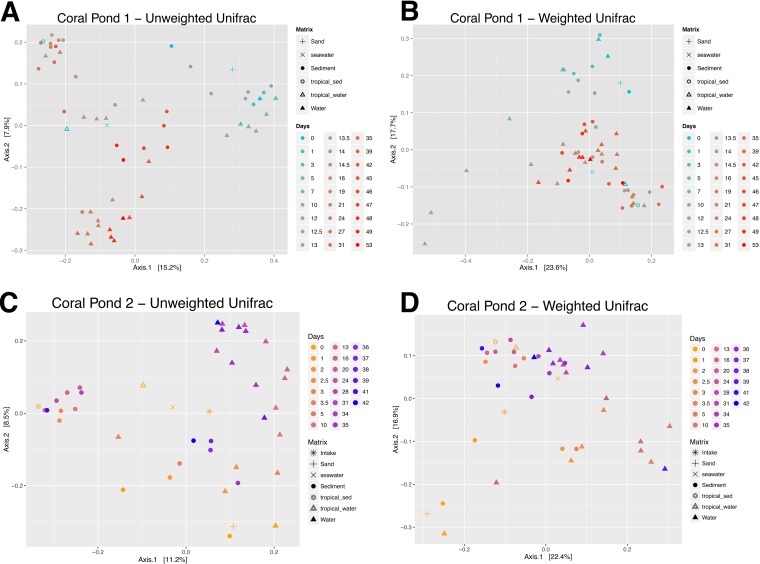
Weighted and unweighted UniFrac PCoA ordination plots showing water, sediment, and perturbation/intake samples from the coral pond 1 (CP1) and coral pond 2 (CP2) experimental aquaria. Principal-coordinate-analysis (PCoA) ordination plots based on UniFrac distances of microbial community 16S rRNA gene profiles display differentiation of microbial communities in sediment (circles) and water samples (triangles) across two independent aquaria (panels A and B, CP1; panels C and D, CP2). Unweighted UniFrac (A and C) and weighted UniFrac (B and D) data are shown. For reference, plots include initial samples from laboratory seawater (“seawater” for CP1 setup and “intake” for CP2 setup) and commercial sand (“sand”) used to set up both aquaria. All plots additionally show sediment (“tropical_sed,” open circles) and water samples (“tropical_water,” open triangles) taken from an established tropical aquarium and used to inoculate CP1. All PCoA ordination plots display results from 97% open-reference OTU picking in QIIME v1.8 (singletons discarded), rarefied at 1,000 sequences per sample.

As expected, sediment and water samples generally showed distinct groupings in unweighted UniFrac analysis (Fig. 6A and C). These results suggest the presence of distinct microhabitats harboring unique microbial assemblages, despite alpha diversity patterns indicating similar high-level taxonomies in water and sediment locations (Fig. 2). Thus, differences in microbial community fingerprints appear to exist at the OTU level, corresponding to lower-level microbial taxonomy (presence/absence of certain families or genera, leading to a distinct mix of phylogenetic lineages being represented in a particular tank location and time point). Commercial sand used for initial aquarium setup displayed a microbial community that was similar to the communities in all CP1 and CP2 samples collected during the first few days of the time series (days 0 to 2; Fig. 6), indicating that the new tanks maintained a “residual” signature of nonnative taxa that was later erased as saltwater aquaria were conditioned and microbial community succession occurred. Similarly, the water and sediment samples collected from an established tropical aquarium (open triangles and circles in Fig. 6, respectively) grouped more closely with the postperturbation aquarium samples, indicating that live rock biofilms and sediment transfers introduced a microbial community fingerprint that was indicative of an established and functional (in terms of nutrient cycling) saltwater aquarium.

The time series of CP1 sediment samples provides a more detailed view of this microbial community succession (Fig. 4). Initially, preperturbation CP1 sediment samples grouped strongly together in the first 12 days of aquarium setup, with the microbial community composition resembling that of the commercial sand. However, after the addition of live rock and transferred sediment on day 12, the microbial communities in CP1 sediments showed an immediate (within a few hours) and rapid shift. By the next sampling event in late afternoon, the CP1 sediment microbial communities showed more similarity to the sediment microbial communities from the established tropical aquarium (CP.12.sed.PM; Fig. 4). This strong grouping was subsequently maintained over time (days 12 to 42), suggesting that this initially rapid shift in microbial community composition represented a jump to a new, stable microbial community profile. However, the final major tank perturbation (the addition of live rocks on day 45) appeared to disrupt this stable successional stage, prompting another microbial community shift toward a third discrete grouping of samples (CP.45.sed; Fig. 4), which was maintained until the end of the experiment. These general microbial community patterns were consistent in CP2 sediments, albeit they were somewhat looser due to the compressed timeline for aquarium setup and perturbation (Fig. 6C). The three distinct groupings of CP1 sediment samples did not demonstrate any obvious associations with the water chemistry measurements, with the exception of high nitrite measurements which appeared to be correlated with the preperturbation microbial assemblages present in CP1 sediments on days 0 to 12 (Fig. S4). It is likely that the microbial taxa that persisted in CP1 sediment prior to day 12 represented a mixture of species that are able to tolerate the rising levels of nitrite and other toxic compounds which were building up in the aquarium due to a nonoptimized nitrification regime.

10.1128/mSphere.00043-19.4 FIG S4 Arrow plot of sediment microbial community profiles based on 16S rRNA gene analysis from the coral pond 1 (CP1) experimental aquarium, showing correlation with water chemistry data. Principal-coordinate-analysis (PCoA) ordination and arrow plots were generated using the vegan package in R. Metadata vectors were generated using “envfit” function in vegan and visualized using ggplot. Download FIG S4, EPS file, 0.02 MB. Copyright © 2019 Bik et al. 2019 Bik et al. This content is distributed under the terms of the Creative Commons Attribution 4.0 International license.

The resilience (rate of recovery) and resistance (tolerance and insensitivity) of community assemblages to disturbance events represent driving issues in microbial ecology (36). A related but distinct concept is that of successional changes in microbial communities and how biological interactions, evolutionary processes, and environmental conditions can reorganize microbial assemblages over time (37). In both CP1 and CP2, the temporal shifts in microbial community structure (richness and diversity) and function (increase in relative abundance of nitrogen-transforming taxa) showed a clear signature of microbial succession. Primary succession occurred following the first perturbation on day 12, where a specially adapted community of saltwater microbes (present on live rock biofilms and transferred sediment particles) colonized new tank habitats that were relatively devoid of biological activity. The second perturbation on day 45 may be indicative of secondary succession, where the disruption to the aquarium ecosystem was severe enough to reshape the microbial assemblages present in a previously colonized habitat. At minimum, this second perturbation event represented a disruption to the equilibrium state (36) and emergence of a new microbial community fingerprint (Fig. 4), although it was unclear whether this shift toward an alternative stable state on day 45 was permanent or temporary. Microbial communities in natural ecosystems have been shown to quickly recover from severe “pulse” disturbance events (36), most notably demonstrated by artificial mixing experiments carried out in temperate lake ecosystems (27, 36). However, such recoveries are not immediate, and the return to predisturbance microbial assemblages in temperate lakes reported in reference 27 was not observed until 7 to 11 days after the artificial disturbance. Given that our sample collection ended 8 days after the second live rock perturbation on day 45, it is possible that our experimental timeline did not adequately capture the full recovery (and shift back to a predisturbance state) of aquarium microbial communities.

**FIG 7:**
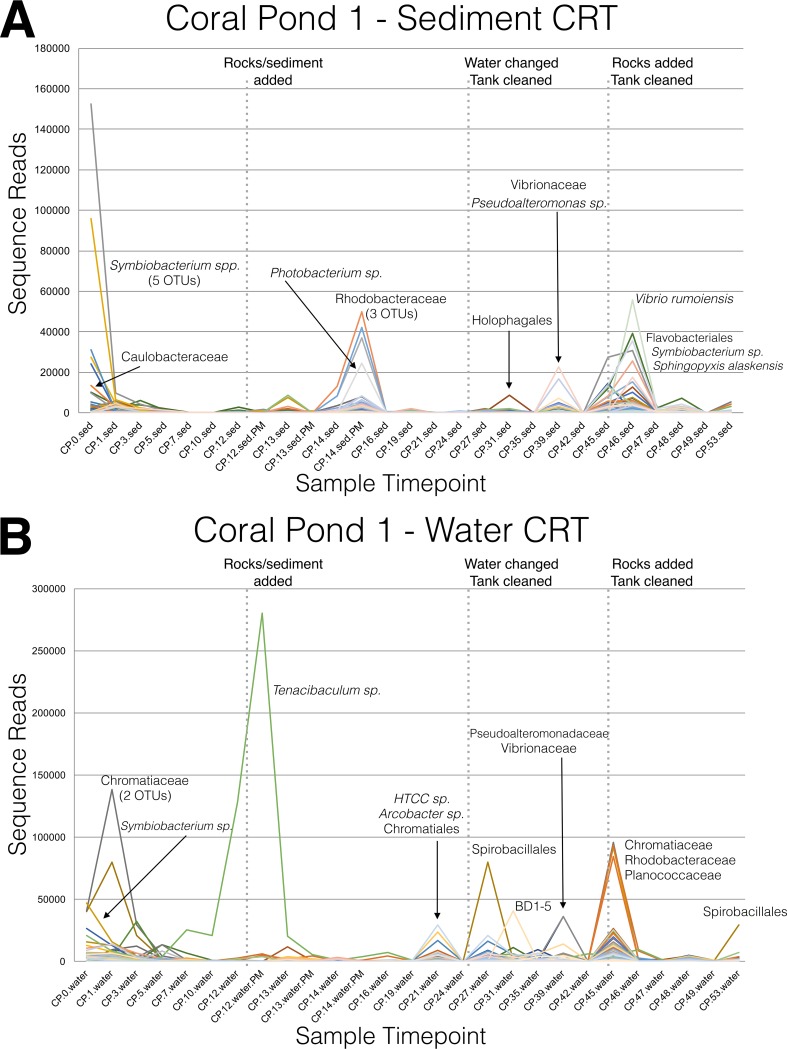
Changes in conditionally rare taxa (CRT) over time in the coral pond 1 (CP1) experimental aquarium. CRT were identified by computing the coefficient of bimodality to statistically detect “blooms” of rare OTUs which were otherwise present at low or zero abundances at most time points, following previously described methods (38). Each colored line represents sequence reads from a single OTU, plotted over time. Taxonomic annotations are indicated for a subset of CRT OTUs with the highest number of sequence reads, with all taxonomic names directly derived from the Greengenes database. Figure panels represent consecutive sample time points collected from sediment (A) and water (B) locations in the CP1 experimental aquarium.

Taken together, our data indicate that live rock additions and perturbations in CP1 and CP2 spurred successional changes that established a microbial community assemblage characteristic of a stable and adequately conditioned saltwater aquarium. Although microbial community profiles of “functional” aquaria have not been previously studied in detail, the perturbations used in the present study represented standard practices in the aquarium trade industry. Thus, our tank setup practices were likely selecting for preferred microbial taxa (e.g., species that can survive in a mesocosm and effectively eliminate compounds that are toxic to larger fish and invertebrates), leading to stable and acceptable water chemistry measurements following tank perturbations and cleaning. The first addition of live rocks and sediment from an established tank on day 12 was especially critical for converting a nonoptimized nitrification regime (e.g., slow buildup of toxic ammonia, nitrite, and nitrate; Fig. 1) to a more desirable and chemically stable cycle. Once the experimental aquaria were conditioned on day 12, the levels of toxic nitrogen compounds were not observed to rise at any subsequent point in the study, despite the continuation of routine perturbations and some observed fluctuations in the relative abundances of nitrogen-transforming taxa (Fig. 5; see also Fig. S2). Thus, both the CP1 and CP2 aquaria appeared to be resistant to disturbance once they gained a specially adapted assemblage of saltwater microorganisms. The nitrogen cycling regimes remained stable in both tanks despite the shift to an alternative stable state following the second live rock perturbation (CP.45.sed; Fig. 4) and the periodic increases in the relative abundances of certain microbial groups and OTUs (Fig. 2 and 7). These results indicate that “healthy” saltwater aquaria may not be characterized by one strictly defined set of microbial taxa but rather that they can regularly shift among multiple stable microbial community states while maintaining a functional nutrient cycling regime. Furthermore, a stable aquarium can routinely exhibit unexplained spikes in microbial abundances without any detrimental impacts on water chemistry or fish and invertebrate health.

### Conditionally rare taxa (CRT) identified across longitudinal data.

As an alternative way of examining microbial community shifts, we implemented an approach using identified conditionally rare taxa (CRT [38]) in searches for statistically significant enrichment of microbial OTUs (taxonomic “blooms”) across longitudinal data sets. Four independent CRT analyses were conducted on both sediment and water sample time series collected from both CP1 and CP2 aquaria using filtered OTU tables specific to each aquarium and sampling location (Fig. 7; see also Fig. S5). Blooms of CRT were sometimes (but not always) correlated with aquarium perturbation events, and many CRT appeared to stochastically increase in relative abundance at isolated time points. The first addition of live rocks and sediment on day 12 did not result in a notable bloom of CRT in CP1 (Fig. 7), despite the dramatic shifts in the overall abundances of major microbial groups (Fig. 2). However, a large number of CRT OTUs were observed in CP1 after the second addition of live rocks on day 45 (Fig. 7), suggesting a destabilizing effect on the overall aquarium microbial assemblages. The abundances of the CRT taxa identified in CP2 samples also seemed to fluctuate more and to exhibit somewhat stochastic patterns during the entire course of the time series sampling, possibly as a result of the compressed timeline corresponding to for CP2 tank conditioning and perturbations.

10.1128/mSphere.00043-19.5 FIG S5 Changes in conditionally rare taxa (CRT) over time in the coral pond 2 (CP2) experimental aquarium. CRT identified by computing the coefficient of bimodality to statistically detect “blooms” of rare OTUs which are otherwise present at low or zero abundances at most time points, following the methods of (38). Each colored line represents sequence reads from a single OTU, plotted over time. Taxonomic annotations are indicated for a subset of CRT OTUs with the highest number of sequence reads, with all taxonomic names directly derived from the Greengenes database. Figure panels represent consecutive sample time points collected from sediment (A) and water (B) locations in the CP2 experimental aquarium. Download FIG S5, PDF file, 0.5 MB. Copyright © 2019 Bik et al. 2019 Bik et al. This content is distributed under the terms of the Creative Commons Attribution 4.0 International license.

The ecological interpretation of CRT (Fig. 7; see also Fig. S5) is complicated by the vagueness of many taxonomic assignments. For example, many OTUs had high-level taxonomic assignments to large and diverse microbial groups (e.g., *Alphaproteobacteria* and *Rhodobacteraceae*), as well as novel lineages such as “J115,” “BD-1,” and “HTCC,” which are listed in the Greengenes taxonomy but not formally recognized within international bacterial taxonomy classification schemes. A number of bacterial groups recovered as CRT are known to contain pathogenic species, including *Photobacterium*, *Vibrionaceae*, *Tenacibaculum*, *Arcobacter*, *Legionellaceae*, and *Bacillus* (39–44). The genus *Tenacibaculum* in particular is of notorious concern within the aquarium trade industry, containing several pathogenic species that widely infect both fish and invertebrates (45, 46). However, the conserved nature of the 16S rRNA gene limits our ability to definitely identify pathogenic versus nonpathogenic bacterial species, especially in some large genera, such as *Bacillus*, where all species display extremely high levels of rRNA gene sequence similarity (47, 48). Some of the identified CRT may play important roles in nutrient cycling and the breakdown of toxic compounds in aquaria. In terrestrial soils, some *Photobacterium* species are rhizosphere-associated symbionts that play a key role in nitrogen cycling (49). The family *Rhodobacteraceae* is comprised of several hundred described species, including many marine members, and phylogenomic evidence suggests that this group plays an important role in diverse biogeochemical cycles (50). The family *Vibrionaceae* and genus *Pseudoalteromonas* are two other large groups of microbes where some members are involved in biogeochemical cycles, including nitrogen fixation (51). The *Chromatiaceae* is the main family of purple sulfur bacteria (a group of photoautotrophs adapted to life in well-illuminated habitats), and this group also contains nitrifying bacteria (52). We recovered two CRT species of *Limnobacter*, a genus containing known sulfur oxidizers. Furthermore, the genus *Arcobacter,* which significantly increased in abundance after the first perturbation in CP1 water and CP2 sediment, contains both pathogenic and nitrogen-fixing species (53). Other groups of CRT appeared to exhibit unique life history traits, including a number of putative biofilm or alga-associated taxa (*Rhodobacteraceae, Saprospira, Legionellaceae* [50, 54, 55]), as well as the family *Pseudoalteromonadaceae*, which contains a few species known to live on algae (56), and groups with potentially novel metabolic pathways. For example, the order *Holophagales* includes species that possess distinct pathways for metal respiration and breakdown of aromatic compounds and may play an important ecological role in the geochemical cycling of metals in the environment (57, 58). Finally, the disappearance of CRT shortly after initial tank setup may indicate that they were nonadapted taxa that could not survive in a saltwater aquarium habitat, such as thermophilic *Symbiobacterium* species (59), which quickly dropped in abundance in CP1 sediment and water (Fig. 7).

Unfortunately, our 16S rRNA gene-based approach does not provide any definitive information about metabolic capability or genomic potential of aquarium microbial communities, and so the cause of CRT blooms and their ecological significance remain unknown. Tools such as PICRUSt (60), Tax4Fun (61), and FAPROTAX (62) can be used to predict the metabolic potential of microbial communities using 16S rRNA gene data. However, such predictive algorithms are inherently limited by the sparse nature of public genome databases (compared to the known levels of diversity) and do not take into account the flexible composition of microbial genomes and strain-level diversity in gene function (63). Given the limitations of these algorithms, and the absence of shotgun metagenome data generated during this study, we have not attempted to make further functional predictions on the basis of analysis of 16S rRNA genes. However, the significant patterns in conditionally rare taxa (and the potential ecological roles of these taxa discussed above) provide an ideal starting point for hypothesis generation and future targeted approaches (e.g., quantitative PCR [qPCR] of known functional genes) or culture-based studies of aquaria.

### Microbial OTUs associated with nitrification.

The vast majority of putative nitrogen-transforming OTUs did not appear as sudden “blooms” and thus did not meet the statistical abundance thresholds required for their identification as CRT. However, there were clear patterns of increased abundance following major aquarium perturbations in CP1 (Fig. 5) and CP2 (Fig. S2). Both tanks contained six major groups of microbial OTUs previously implicated in the cycling of nitrogenous compounds such as ammonia, nitrite, and nitrate in aquarium systems. A total of 552 OTUs were assigned to the Thaumarchaeota phylum of Archaea, which contains all known AOA (64), with 346 of these OTUs classified in the *Nitrosopumilus* genus. Two major groups of AOB were detected: *Gammaproteobacteria* of the genus *Nitrosococcus* (2 OTUs) and *Betaproteobacteria* of the family *Nitrosomonadaceae* (106 OTUs in total, with 7 of these OTUs assigned to the well-described ammonia-oxidizing species *Nitrosovibrio tenuis* [65]). A large number of OTUs were also associated with *Planctomycetes* (3,635 OTUs classified under class *Planctomycetia*, including assignments to the marine anammox genus *Scalindua* (21), despite the vast majority of *Planctomycetia* OTUs remaining unclassified at the genus or species level. Finally, we detected two major groups of NOB: *Deltaproteobacteria* assigned to the genus *Nitrospina* (169 OTUs) and OTUs in the phylum *Nitrospirae* (class *Nitrospira*; 450 OTUs mostly assigned to the family *Nitrospinaceae*). Most nitrogen cycle-associated taxa showed a marked increase in relative abundance following the first major aquarium perturbation. This change was best illustrated in CP1 water and sediment (Fig. 5), where Thaumarchaeota, *Nitrospira*, and *Scalindua* OTUs showed the most obvious increases in relative abundance, along with *Nitrosomonadaceae* and *Nitrospina* OTUs to a lesser extent. The increased relative abundances of these taxa appear to be indicative of an aquarium with a satisfactory nitrification regime: postperturbation levels of nitrogen-transforming OTUs in CP1 and CP2 were comparable with the relative abundances of these OTUs in the established tropical saltwater aquarium (T.0.water and T.0.sed samples in Fig. 5 and Fig. S2), and these nitrogen-transforming taxa have been previously found to be associated with both saltwater and freshwater aquaria (17, 26, 66–68).

Nitrogen cycling in saltwater aquaria is comparatively understudied compared to what is known about nitrification in freshwater aquaria (25, 26, 66, 67) and other aquatic systems such as bioreactors and wastewater treatment (19, 69). The microbial species responsible for nitrification appear to be distinct between saltwater and freshwater aquaria (66), and denaturing gradient gel electrophoresis (DGGE) performed with archaeal *amoA* genes indicated a separation of community profiles between freshwater and saltwater aquaria (24). To the best of our knowledge, anammox organisms have not been previously reported in freshwater or saltwater aquaria. Our data set contains OTUs assigned to *Scalindua* species, the only known marine group of anammox bacteria that are typically found in anaerobic sediments (e.g., deep-sea methane seeps [70]) or oxygen-poor waters (e.g., oxygen minimum zones [71]). The relative abundances of *Scalindua* OTUs increased after the first aquarium perturbation on day 12, along with increases in the levels of other nitrogen-transforming OTUs (Fig. 5). However, oxygen levels in both the CP1 and CP2 aquaria remained high and relatively constant (7 to 8 mg/liter; Fig. 1) throughout the course of the experimental time series, suggesting that anammox *Scalindua* species could be inhabiting oxygen-poor microhabitats within aquaria (e.g., within biofilms or within the sediment). Taken together, these results suggest that the phylogenetic and functional diversity of nitrogen-transforming taxa in saltwater aquaria is likely to be far greater than currently recognized, and further research is needed to elucidate the biodiversity and metabolic capabilities of key microbial taxa involved in biogeochemical cycling in marine ecosystems.

### Conclusions.

This report provides a detailed view of the fine-scale patterns of microbial community succession and response to perturbation in two saltwater aquaria. Our data set provides an overview of community changes over time, including the impacts of aquarium setup, conditioning, and routine tank maintenance such as wall cleaning and water changes. Notably, our results suggest that changes in microbial community composition do not always correlate with water chemistry measurements, and some functionally important OTU-level patterns (e.g., postperturbation increases in the levels of nitrogen-transforming taxa and recovery of anammox *Scalindua* OTUs) were not reported as statistically significant. The ecological relevance of conditionally rare taxa was unclear, despite these OTUs showing statistically significant increases in relative abundance over time. Furthermore, successional patterns indicate the existence of multiple “stable states” for aquarium microbial assemblages, where routine tank disturbances can prompt shifts between different microbial stable states without any increases in the level of toxic nitrogenous compounds (ammonia, nitrite). Future studies of aquaria should also aim to characterize functional changes that occur over time using metagenomics (e.g., assessing enrichment or depletion of genes involved in nutrient cycling processes) and to utilize narrower experimental approaches to quantify the effects of specific perturbations. Aquaria represent an ideal mesocosm system that can be easily leveraged to test diverse biological hypotheses. Such studies are broadly applicable to our understanding of marine and freshwater ecosystems, while also deepening our knowledge of engineered environments such as aquaculture systems, where nitrogen cycling regimes are critical for the production of food and biofuels.

## MATERIALS AND METHODS

### Experimental design and sample collection.

The two “coral ponds” described in this study (CP1 = coral pond 1; CP2 = coral pond 2) were assembled in fall 2012 at the University of California, Davis (UC Davis). Aquaria consisted of a pair of large (∼900-liter) plastic containers that were filled to a depth of ∼30 cm with commercial sand (coral sand, size 0; Aquaglobe, Campbell, CA, USA), followed by addition of ∼375 liters of seawater each from the UC Davis distribution system (origin, Bodega Bay, CA). These two aquaria were being assembled in the UC Davis teaching laboratories for use in undergraduate biology courses, and our rationale for focusing on these two tanks was based on convenience, proximity, and familiarity for undergraduate students involved in sample collection and data analysis. At specific time points (see Fig. 1), the following items were added to each aquarium: a protein skimmer (“Remora Pro” model; AquaC, Rock Hill, SC), lighting/heating (DA Luminaire Services DAUN lamp unit; Hydrofarm, Petaluma, CA) with a True 14,000-Kä bulb (Hamilton Technology, Gardena, CA), sediment and coral from an existing aquarium (an established “tropical aquarium”; see below), and cured live rocks (purchased from an unknown aquarium supplier). Each aquarium underwent the following three major perturbation events that disrupted the water and sediment within each tank: a first perturbation (day 12) comprising an addition of live rocks and sediment transferred from the tropical aquarium, a second perturbation where water replacement and wall cleaning were performed (day 26 for CP1, day 28 for CP2), and a final perturbation where an additional set of live rocks were added at the same time that the tank walls were cleaned (day 45). Prior to their placement in experimental aquaria, live rocks were kept in barrels for approximately two months, with occasional water changes in order to “cure” them. Live rocks are typically collected from coral reefs and packed in wet newspaper for shipment around the globe, resulting in a large number of dead or dying organisms within the rock structure (72); the “curing” process is thus necessary to restabilize the live rock microbial communities and remove waste products resulting from organic matter degradation before live rocks are added to a new aquarium. During tank maintenance, water was replaced by removing approximately half the volume of water in each aquarium and transferring an equivalent volume of water into each aquarium from the UC Davis building seawater distribution system (origin, Bodega Bay, CA); the walls of each aquarium were simultaneously cleaned by removing visible algae with clean cloth wipes. Samples were collected from CP1 and CP2 aquaria over a three-month period from 30 November 2012 to 22 January 2013, and the frequency of sampling was increased around the perturbations. These aquaria were being assembled by university personnel in preparation for classroom use; therefore, the timing of tank setup, conditioning, and perturbations was predetermined by course needs. In addition to the CP1 and CP2 tanks, samples were also collected from an established tropical aquarium at UC Davis in order to capture microbial community profiles in a longer-term stable tank setup; the tropical aquarium consisted of a rectangular, glass-walled saltwater tank with a water volume of approximately 100 gallons. Sediment, water, and wall samples from the tropical tank were collected in the same manner as was used for the CP1 and CP2 aquaria (the methods used are described below).

At each time point, triplicate samples for DNA sequencing were collected from each of three locations (i.e., water, sediment, and tank walls) in both aquaria. Additional water samples were collected from each aquarium for water chemistry analysis at every time point. Sediment samples were collected by transferring the top 5 cm of sediment into 1.5-ml microcentrifuge tubes. During initial tank setup (day 0), additional “commercial sand” samples (CP.0.sand, CP2.0.sand) were collected from the dry bags of sediment used to establish the CP1 and CP2 aquaria using the same sampling procedure as was used for the tank sediment samples. Water samples were collected from the middle of the water column with a freshly rinsed 1-liter flask, and this volume was subsequently vacuum filtered onto 0.1-μm-pore-size filters (Supor PES membrane disc filters; Pall Corporation, Port Washington, NY). During the initial tank setup (day 0), samples were also collected from the building seawater system (IW.0 water, CP2.0.intake); the building seawater tap was run for 1 min before sample collection in order to purge stagnated water, and seawater samples were collected via vacuum filtration using the protocol described above. Microbial communities on aquarium walls were also collected using vertical swab sampling (spanning the full aquarium length from water surface to sediment), using one-quarter of a standard Kimwipe (Kimtech Science KimWipes Delicate Task Wipers, catalog no. KIM34155; Kimberly Clark, Irving, TX, USA), and then frozen. All samples were initially frozen at −20°C for temporary storage and later moved to −80°C for long-term storage after DNA extractions and PCR were completed. Due to low DNA yields and inconsistent PCR amplifications, the aquarium wall samples were disregarded in the present study and were not subjected to further sequencing and analysis. Samples collected from the tropical aquarium, commercial sand, and building seawater system were subjected to DNA extraction and PCR amplification using the same protocols as were used for the CP1 and CP2 experimental tank samples, and all project samples were pooled for Illumina sequencing and downstream bioinformatics analysis (the methods used are described below).

During every sampling event, the following water chemistry measurements were collected: pH (Pinpoint pH Monitor Package; American Marine Inc., Ridgefield, CT, USA), salinity (measuring percent salt [refractometer] values) (Pinpoint salinity monitor; American Marine Inc.), temperature (Pinpoint wireless thermometer; American Marine Inc.), hardness (Hanna Instruments HI 3817 water quality test kit; Hanna Instruments, Woonsocket, RI, USA), alkalinity (HI 3817 water quality test kit; Hanna Instruments), chloride (HI 3817 water quality test kit; Hanna Instruments), sulfide (HI 3817 water quality test kit; Hanna Instruments), dissolved oxygen (HI96732 dissolved oxygen portable photometer; Hanna Instruments), ammonia (measuring NH_3_-N-free ammonia nitrogen levels; HI96715 waterproof portable medium-range ammonia photometer; Hanna Instruments), nitrate (HI96786 Cal Check nitrate portable photometer; Hanna Instruments), nitrite (measuring NO_2_-N nitrite-nitrogen; HI-764 Checker HC handheld colorimeter for nitrite; Hanna Instruments), and phosphorus (HI-736 Checker HC handheld colorimeter for phosphorus; Hanna Instruments). All kits and instruments used for water chemistry measurements were obtained via Amazon.com or other online specialist suppliers of aquarium systems.

### DNA extraction.

DNA extractions were performed using a Mo Bio PowerSoil DNA isolation kit (catalog no. 12888-100; Mo Bio Laboratories Inc., Carlsbad, CA, USA). Sediment was mixed in the collection tube using a flame-sterilized metal spatula, and then a pea-sized scoop was transferred to the PowerBead tubes. Water filter halves were cut into small pieces using flame-sterilized scissors and then put into PowerBead tubes. The standard extraction protocol was carried out with the following changes: 10 min of vortex mixing was followed by 60 s of beating using a bead beater (BioSpec Products, Bartlesville, OK, USA) at maximum speed in order to improve lysis of spores and tough bacterial cell walls and reduce DNA shearing, and the final elution step was carried out using 50 µl of solution C6 instead of 100 µl. Once extracted, DNA was quantified using a high-sensitivity Quant-iT double-stranded DNA (dsDNA) assay kit (catalog no. Q33120; Life Technologies, Carlsbad, CA, USA) and a Qubit fluorometer (catalog no. Q32857; Invitrogen, Carlsbad, CA, USA). One “blank” DNA extraction, where molecular-grade water was used as an input instead of any sample material, was also performed as a negative control; that kit control sample was subjected to downstream PCR amplifications and Illumina sequencing alongside the aquarium samples.

### 16S rRNA gene library construction and sequencing.

Marker gene libraries were generated from aquarium DNA extractions using one of two library construction protocols (depending on the availability of reagents): one using DNA *Taq* polymerase (catalog no. 201203; Qiagen, Hilden, Germany) and the other using Kapa HotStart *Taq* DNA polymerase (kit code KK1509; Kapa Biosystems, Wilmington, MA, USA). A two-step PCR protocol was used as follows. During the first round of PCR (PCR A), 16S rRNA genes were amplified from environmental DNA, and during the second round of PCR (PCR B), the partial adaptor sequence attached to PCR A primers was extended into the full-length Illumina sequencing adaptor. Dual-index barcoded primer sets and adaptor extension primers were purchased from Invitrogen (Carlsbad, CA) (the full sequences of all primer constructs were previously published by Lang et al. [73]). PCR A used annealing at 52°C for 30 s and elongation at 72°C for 1.5 min, for 10 cycles. PCR B used annealing at 62°C for 30 s and elongation at 72°C for 1.5 min, for 18 cycles. Following both PCR A and PCR B, agarose gel electrophoresis was used to confirm successful PCR amplification, and PCR cleanup was carried out using Agencourt AMPure XP magnetic beads (part no. A63882; Beckman Coulter, Brea, CA, USA) with a 1:1 bead-to-DNA volume ratio. A final DNA quantification step after PCR B was carried out using a Quant-iT dsDNA assay kit and a Qubit fluorometer as listed above. Libraries were normalized to 1 ng per library and pooled in batches of 80 to 100 samples for sequencing. Sequencing was performed on an Illumina MiSeq platform (Illumina) at the UC Davis Genome Center DNA Technologies Core, using either PE250 or PE300 sequencing chemistry. Aquarium samples were sequenced across four MiSeq runs, using dual index barcodes to differentiate samples within each run. A total of 303 aquarium samples were sequenced, including one blank DNA extraction sample included as a kit control. The total number of processed Illumina reads obtained across all samples was 37,556,213 (representing nonsingleton OTUs containing >2 reads; see Table S1 in the supplemental material).

10.1128/mSphere.00043-19.6 TABLE S1 Sequences obtained across all samples in the present study. Table columns list the number of per-sample barcode matches obtained during initial demultiplexing, the number of sequence reads that subsequently passed paired-end merging and quality trimming, and final number of reads retained per sample after 97% open-reference OTU picking in QIIME (summarized for nonsingleton OTUs containing >2 reads). Download Table S1, XLSX file, 0.02 MB. Copyright © 2019 Bik et al. 2019 Bik et al. This content is distributed under the terms of the Creative Commons Attribution 4.0 International license.

### Data processing.

Raw sequence data were demultiplexed and checked for quality using an in-house custom pipeline, *Demul_trim_prep_250.pl* (available on GitHub at https://github.com/gjospin/scripts/blob/master/Demul_trim_prep_250.pl). Default data processing parameters were determined according to quality-filtering recommendations for Illumina data (74). Samples were demultiplexed by matching nucleotide barcodes to Illumina index reads, allowing for one mismatch maximum for each index mate. For quality control, sequence reads were trimmed when the end base reached a minimum score of Q20 moving inward from the end of each read, and the last 50 bases were removed from PE-300 reads because longer reads spanned more than the targeted fragment and extended into the Illumina adapter. Once the reads had been trimmed and subjected to quality control, paired-end reads were merged using FLASH (75) with the read length parameter set as 250 or 300 (corresponding to the Illumina sequencing chemistry used). Minimum overlap was set at 10 bp and maximum at 120 bp. All other parameters were left at the default settings. Paired-end reads that did not merge successfully were discarded. After the reads were merged, the files were reformatted from FASTQ to FASTA, with FASTA headers reformatted to match QIIME requirements (>SampleName_SeqNumber), and the sample identifiers (IDs) were renamed to match the master metadata mapping file. Processed sequence data from the four Illumina runs were concatenated into a single file before QIIME analysis, with renumbering of sequences performed where required for samples that were sequenced across multiple Illumina runs.

### QIIME workflows and statistical analyses.

All downstream data processing was carried out using QIIME version 1.8.0 (76). Paired-end reads that were overlapped and merged were then subjected to a subsampled open-reference OTU picking workflow at 97% pairwise identity (77) using the *pick_open_reference_otus.py* script in QIIME. The OTU picking workflows were carried out using the default UCLUST algorithm and parameters (78), with the addition of the *–enable_rev_strand_match* flag, which allowed input reads to be assessed in both standard and reverse complementation orientations. The level of subsampling was set at 10% (*-s 0.10*). Taxonomy assignments were carried out using the default algorithm (UCLUST consensus taxonomy assigner) and the latest QIIME-formatted release of the Greengenes database collapsed at 97% sequence identity (gg_otus-13_8-release). Chimera checking was run on the resulting 16S rRNA gene OTUs, using the ChimeraSlayer algorithm (79) as implemented in QIIME script *parallel_identify_chimeric_seqs.py*. Flagged chimeras were subsequently filtered out of the OTU table and removed from further downstream analysis.

Following OTU picking, sample replicates were visually compared to confirm that triplicate samples from each sampling location and time point exhibited similar microbial community profiles (i.e., indicating that the participation of different project personnel and undergraduate students did not impact the replicability of aquarium microbial community profiles). Manual assessment confirmed that project staff and PCR protocols did not introduce any obvious downstream bias into the recovery of aquarium microbial communities; the community profiles corresponding to each sample location/time point were replicable and consistent. Subsequently, sample replicates were collapsed into one sample ID representing each aquarium location and time point for all downstream analyses of the diversity of the microbial populations, using the *summarize_otu_by_cat.py* command in QIIME (in addition, the flag *-c collapsereps* was used to reassign sample IDs using the appropriate column in the sample mapping file). This open-reference OTU table with collapsed replicates was subjected to more-stringent filtering based on taxonomy and sequence alignment (see Materials and Methods), which further reduced the number of reads and OTUs used in downstream data visualization and microbial community analysis.

To clean up the data set further, OTU tables were then further filtered by (i) removing sequences that were taxonomically identified as being from chloroplasts and mitochondria, (ii) removing OTUs with taxonomy assignments listed as “unassigned” at the root level, and (iii) removing any OTUs whose representative sequences failed to align to the Greengenes database using the *parallel_align_seqs_pynast.py* script. Beta diversity analyses, including Bray-Curtis, Jaccard, Canberra, and both weighted and unweighted UniFrac metrics analyses, were carried out using the *beta_diversity_through_plots.py* script. The OTU tables were rarefied according to the sample with the lowest sequencing depth, resulting in a rarefaction value that was typically set at 1,000 sequences per sample (a number chosen to avoid the need to discard a number of time series samples with lower sequencing depths).

Water chemistry data, filtered OTU tables, and PCoA distance matrices were imported into Excel or R Studio (R version 3.1.0) for further visualization and exploration of microbial community patterns. Longitudinal samples from CP1 and CP2 (i.e., water and sediment samples) were sorted according to sampling time point in order to assess changes in microbial assemblages over time. The OTU taxonomic assignments were summarized at various levels (e.g., phylum and genus) to visualize broad-scale versus fine-scale community shifts using *summarize_taxa.py* in QIIME. Following the approaches outlined previously by Shade et al. (38), we applied a statistical method for identifying conditionally rare taxa (CRT) in our time series. This CRT approach tracks temporal changes in the relative abundances of 16S rRNA gene OTUs, computing the coefficient of bimodality to identify statistically significant “blooms” of rare OTUs that are otherwise present at low or zero abundances for most time points. For CRT time series analyses, filtered OTU tables were separated and four longitudinal data sets were analyzed independently according to aquarium and sampling location (i.e., CP1 sediment, CP1 water, CP2 sediment, CP2 water). To identify potential environmental drivers of shifts in the diversity of microbial populations, arrow plots were also generated using the vegan package in R. Metadata vectors were generated using the “envfit” function and overlaid onto ordinations that were visualized using ggplot.

### Data availability.

Demultiplexed raw reads were deposited in GenBank (BioProject accession number PRJNA284506) as BioSample accession numbers SAMN03731394 through SAMN03731695. Demultiplexed, trimmed, and merged reads (QIIME ready) have also been deposited on Figshare (https://doi.org/10.6084/m9.figshare.1427397) along with documentation of command line analyses and QIIME parameters employed during the course of this study, as well as the QIIME mapping file listing sample information and water chemistry measurements.
