# Supplementary figures and images for: Microbial Community Succession and Nutrient Cycling Responses following Perturbations of Experimental Saltwater Aquaria
Source: mSphere. 2019 Feb 20;4(1):e00043-19. doi: 10.1128/mSphere.00043-19 (PMC6382968; doi:10.1128/mSphere.00043-19)

# A

## Coral Pond 1

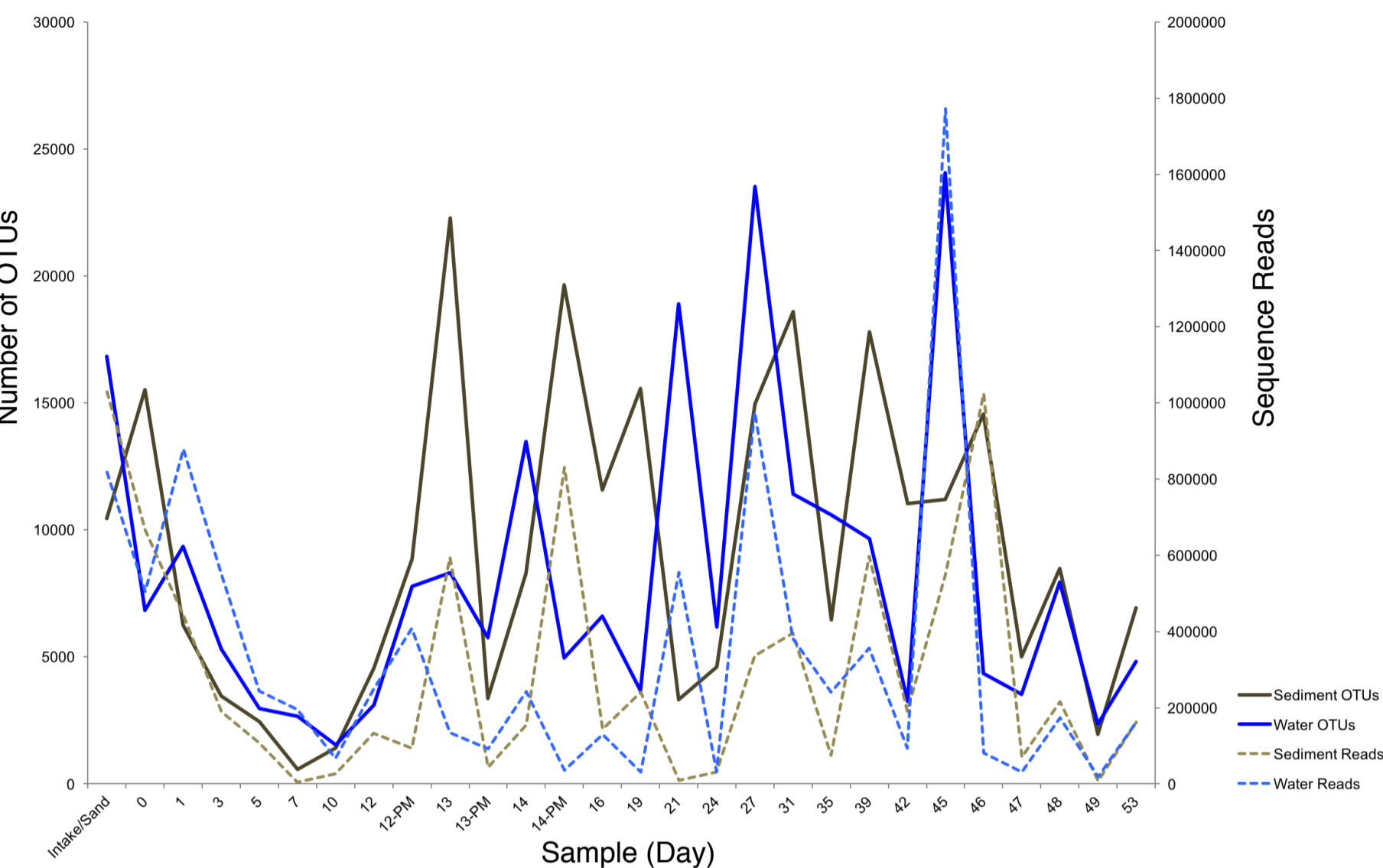

# B

## Coral Pond 2

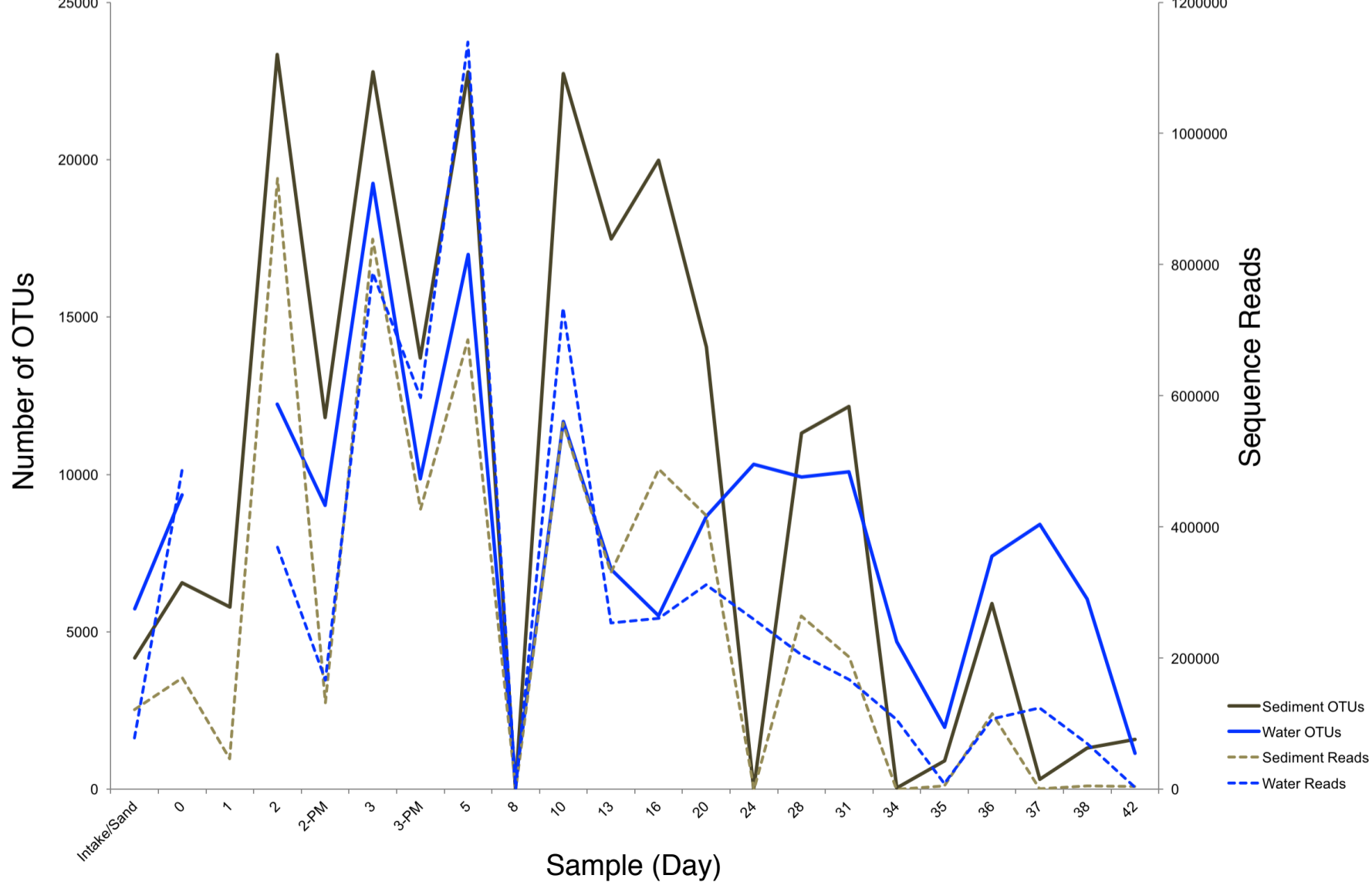

Supplement: FIG S1 [file mSphere.00043-19-sf001.pdf]

**A**

## Nitrogen-transforming OTUs - CP2 Sediment

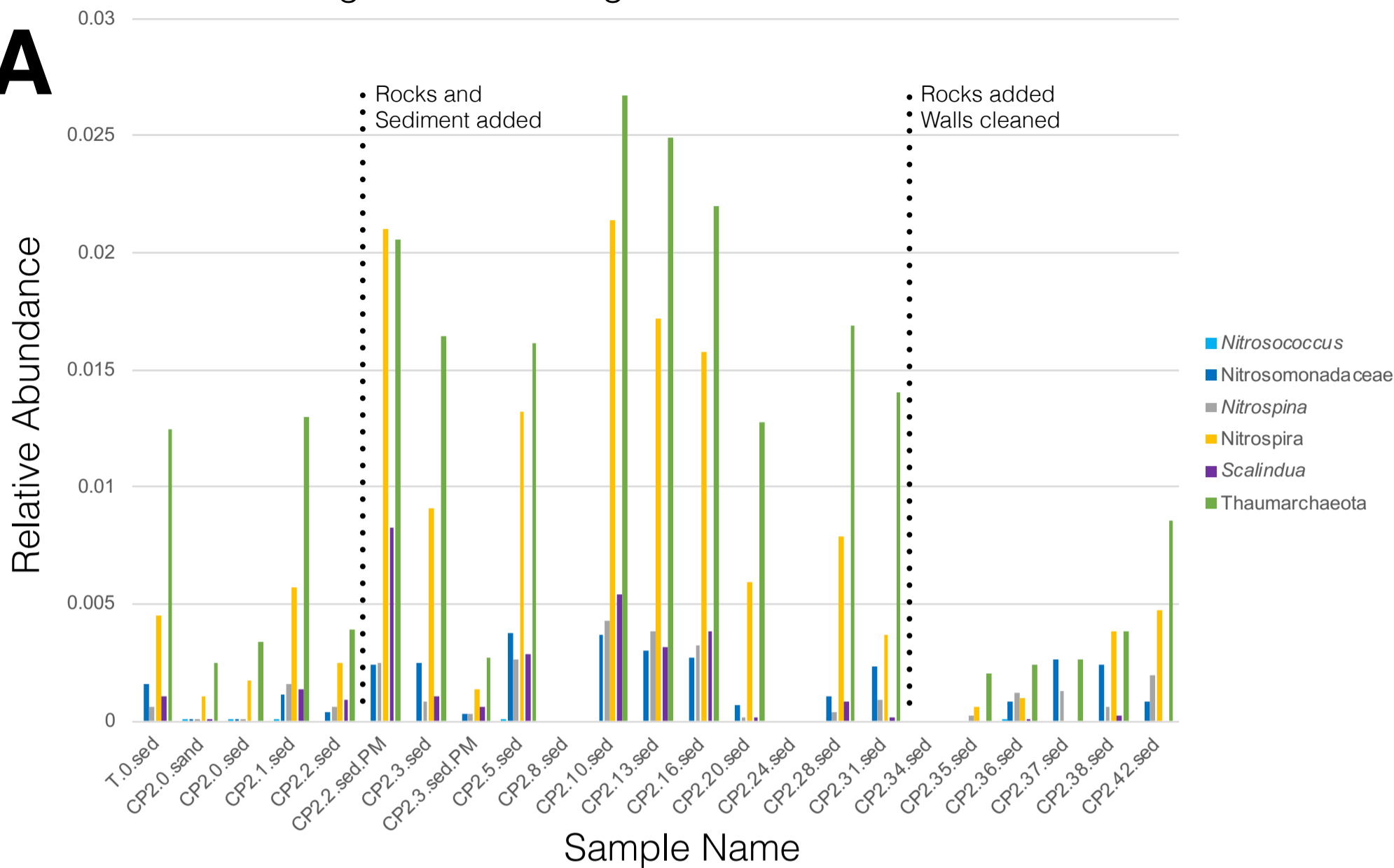**B**

## Nitrogen-transforming OTUs - CP2 Water

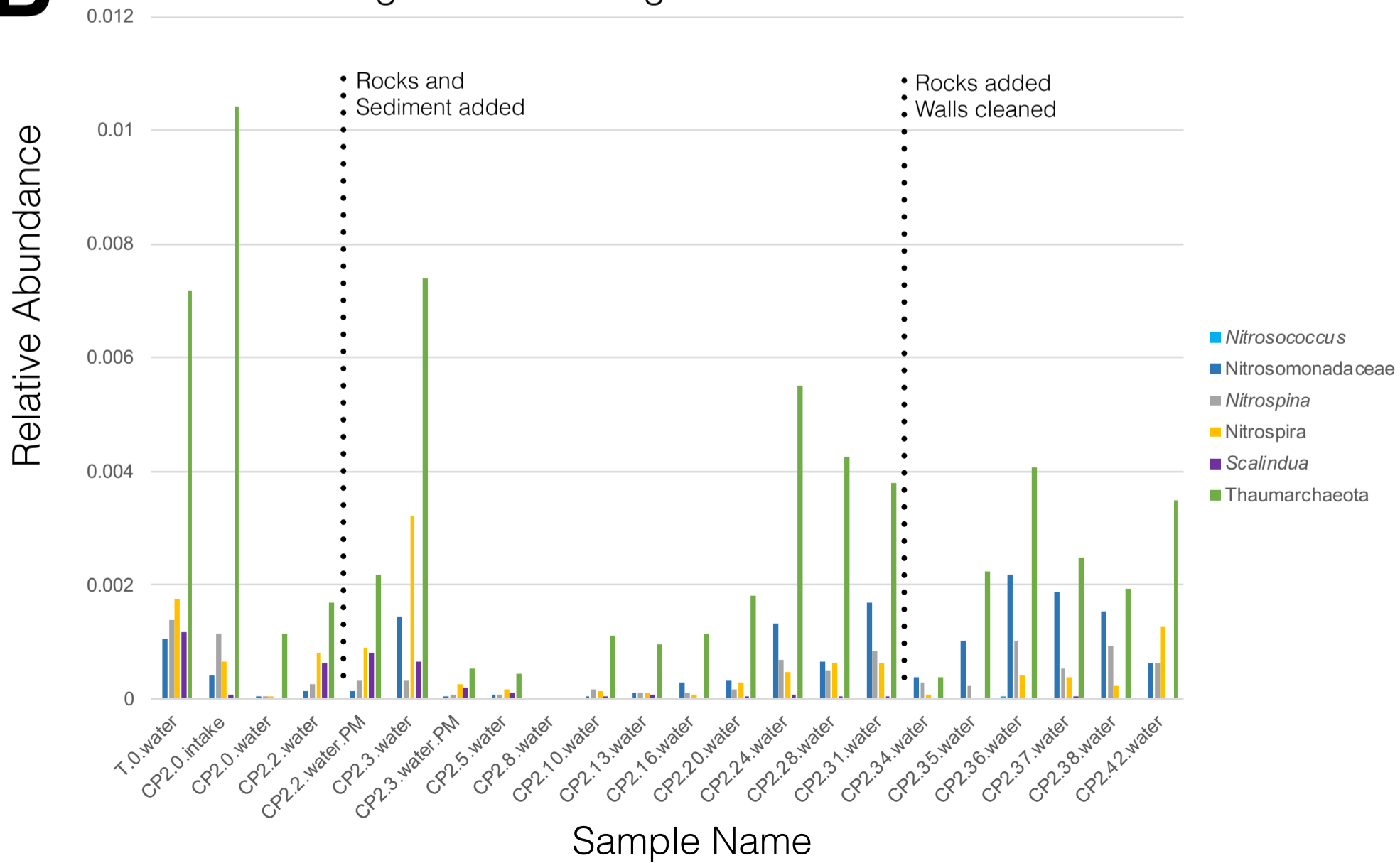

Supplement: FIG S2 [file mSphere.00043-19-sf002.pdf]

# A Coral Pond 2 - Sediment CRT

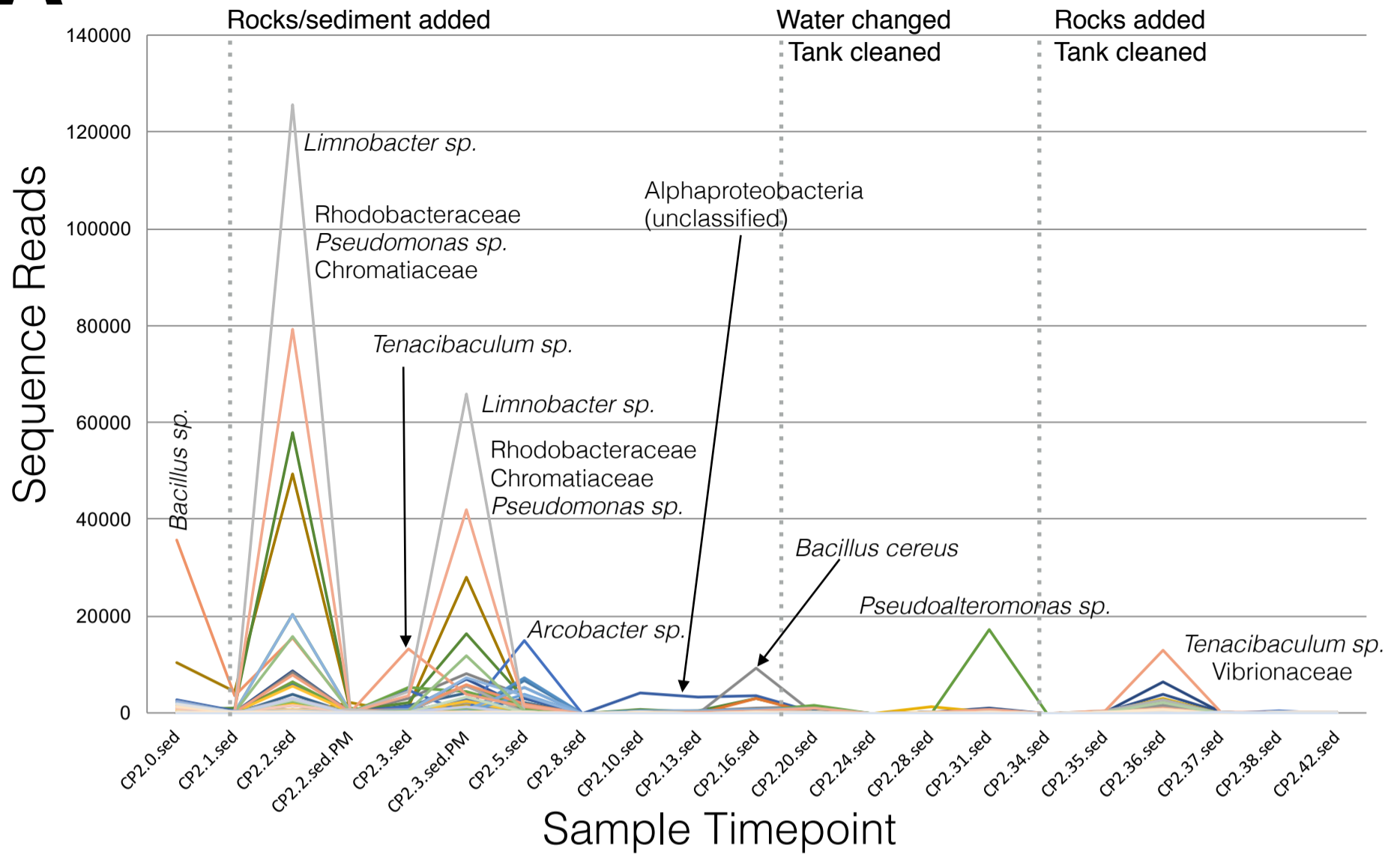

# B Coral Pond 2 - Water CRT

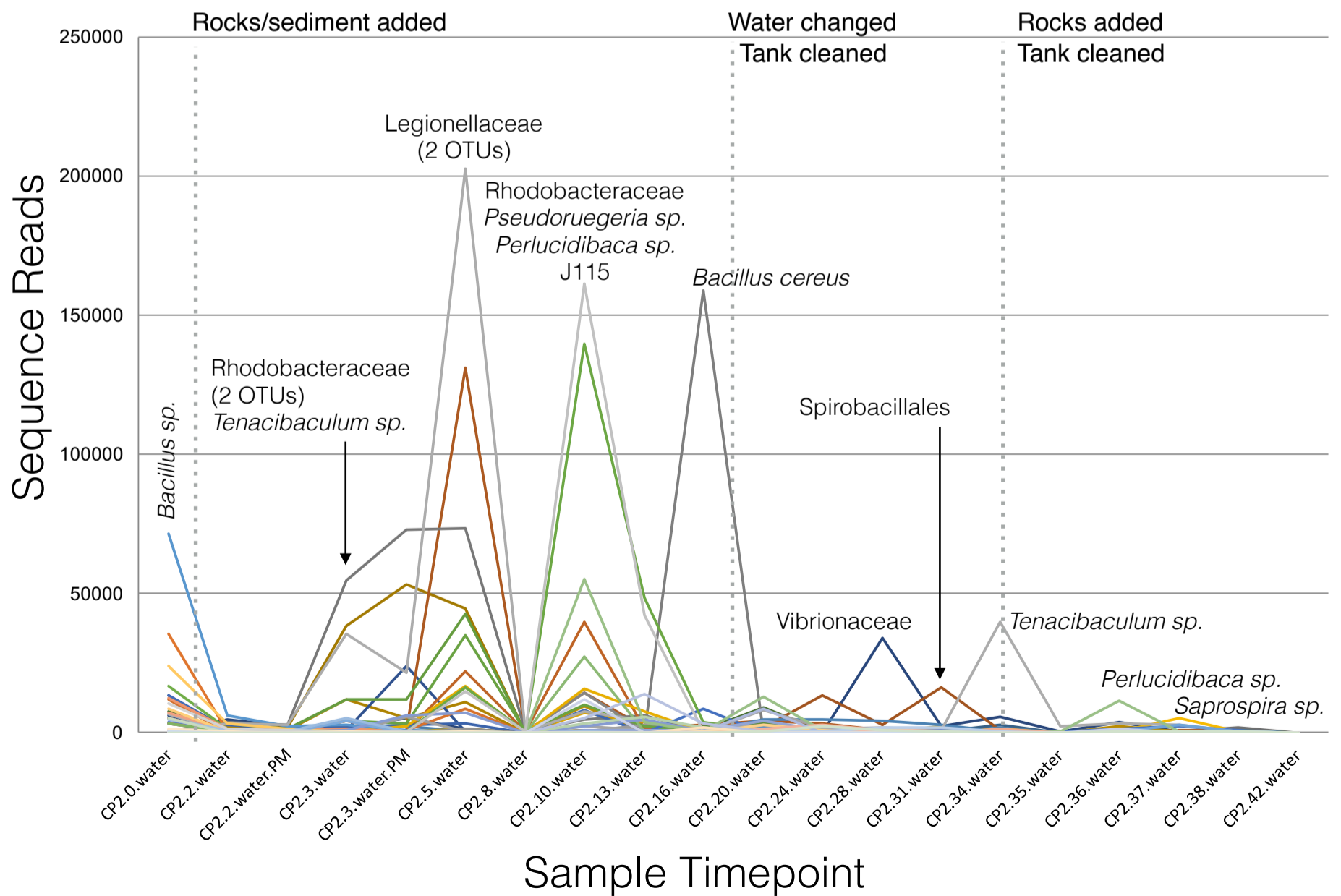

Supplement: FIG S5 [file mSphere.00043-19-sf005.pdf]
